# X-linked inhibitor of apoptosis inhibits apoptosis and preserves the blood-brain barrier after experimental subarachnoid hemorrhage

**DOI:** 10.1038/srep44918

**Published:** 2017-03-22

**Authors:** Cheng Gao, Hongwei Yu, Cong Yan, Wenyang Zhao, Yao Liu, Dongdong Zhang, Jingwei Li, Nan Liu

**Affiliations:** 1 Department of Neurosurgery, The First Affiliated Hospital of Harbin Medical University, Harbin, Heilongjiang, China

## Abstract

Early brain injury following subarachnoid hemorrhage (SAH) strongly determines the prognosis of patients suffering from an aneurysm rupture, and apoptosis is associated with early brain injury after SAH. This study was designed to explore the role of X-linked inhibitor of apoptosis (XIAP) in early brain injury following SAH. The expression of XIAP was detected using western blotting and real-time RT-PCR in an autologous blood injection model of SAH. We also studied the role of XIAP in early brain injury and detected apoptosis-related proteins. The results showed that XIAP was significantly up-regulated in the cortex and hippocampus and that XIAP was mainly expressed in neuronal cells following SAH. The inhibition of endogenous XIAP aggravated blood-brain barrier disruption, neurological deficits and brain edema. Recombinant XIAP preserved the blood-brain barrier, improved the neurological scores and ameliorated brain edema. Recombinant XIAP treatment also decreased the expression of cleaved caspase-3, caspase-8 and caspase-9, whereas there was no effect on the expression of p53, apoptosis-inducing factor or cytochrome c. These results show that XIAP acts as an endogenous neuroprotective and anti-apoptotic agent following SAH. The effects of XIAP on early brain injury was associated with the inhibition of the caspase-dependent apoptosis pathway.

Subarachnoid hemorrhage (SAH) following intracranial aneurysm rupture results in high mortality and morbidity. Despite progress in the clipping and coiling of cerebral aneurysms, no significant improvements have been made in the prognosis for SAH^1^. Early brain injury (EBI), which is defined as primary insults to the whole brain within the first 72 h after SAH, is thought to be an important factor in determining the prognosis of patients with SAH^2^. Apoptosis has been shown to play a pivotal role in EBI both in clinical and experimental settings^3^. Anti-apoptosis treatment has been shown to have neuroprotective effects in an experimental animal model of SAH^4^,^5^. However, systemic anti-apoptosis treatment may have potential side effects because apoptosis is also important for maintaining normal physiological function^5^. Endogenous inhibitors of apoptosis (IAPs) regulate apoptosis by combining with executor caspases and/or initial caspases^6^. X-linked IAP (XIAP) is the most potent inhibitor of apoptosis and is expressed in neuronal cells. When it is combined with caspase-3 and other members of the caspase family, XIAP inhibits apoptosis^7^,^8^. To our knowledge, no studies have examined whether XIAP is altered or contributes to EBI after SAH. In the present study, we investigated the role of the XIAP in SAH-induced brain injury in rats; specifically, we investigated the role of XIAP in BBB disruption.

## Materials and Methods

### SAH model and study protocol

The animal protocols were approved by the Special Committee on Animal Welfare of Harbin Medical University and were conducted in accordance with the guidelines for the Care and Use of Laboratory Animals published by the National Institutes of Health (NIH Publication No. 85-23, revised 1996).

Autologous blood was injected into the prechiasmatic cistern to induce SAH. Male Sprague-Dawley rats (weighing 280 to 320 g) were anesthetized with 2.0% isoflurane in 70% nitrous oxide and 30% oxygen using a face mask. A PE-50 catheter was inserted into the right femoral artery for blood collection and to monitor blood pressure and blood gas. Rectal temperature was kept at 37 °C during surgery using a heating pad. The animals were mounted in the stereotactic frame, and a needle was tilted 60° in the sagittal plane and placed 8 mm anterior to bregma in the midline, with a hole into the prechiasmatic cistern facing the right side. The needle was inserted to the 2 mm anterior to the optical chiasm and 0.3 mL nonheparinized fresh autologous arterial blood was injected into the prechiasmatic cistern in 30 s with a syringe pump. Sham-operated rats underwent identical procedures, except saline was injected instead of autologous blood.

**Figure 1:**
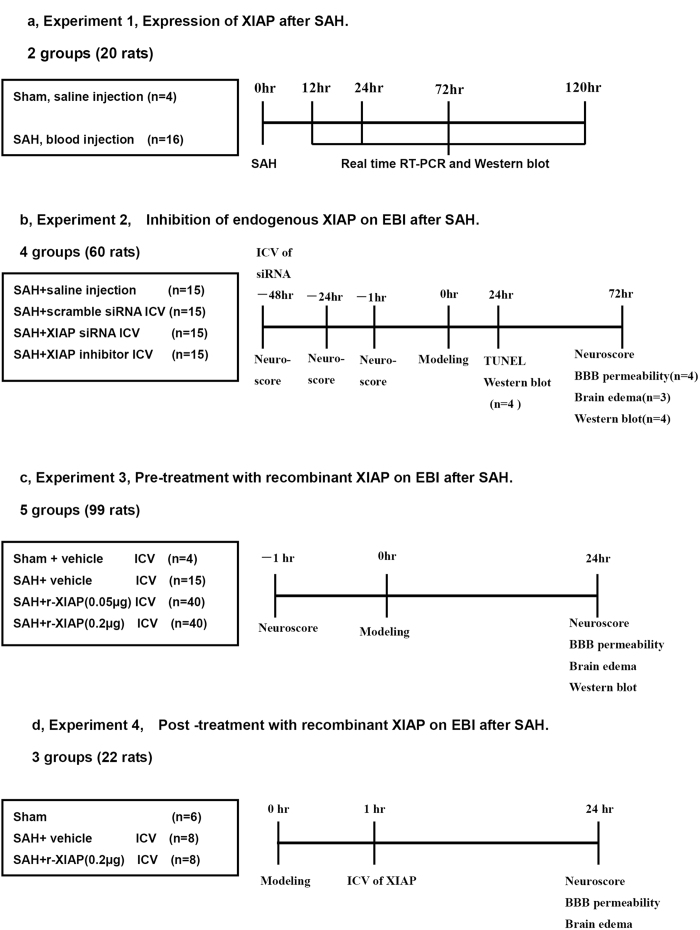
Study design.

First, XIAP expression was assessed at different time points after SAH (6, 12, 24, 72 and 120 h, n = 4 for each time point and n = 4 in the sham control group, Fig. 1a).

Second, we explored the effects of inhibition of endogenous XIAP after SAH on EBI. Rats (n = 60) were randomly assigned to SAH+ phosphate-buffered saline (PBS, n = 15), SAH+ scrambled control siRNA (n = 15), SAH+XIAP siRNA (n = 15) or SAH+XIAP inhibitor (embelin, n = 15) groups. At 24 h following SAH, 4 rats were sacrificed in each group to detect expression of XIAP and apoptosis-related proteins with Western blotting and neuronal apotosis was detected by TUNEL staining. Body weight, neurological scores, BBB permeability and brain water content were detected 72 h after SAH (Fig. 1b).

Third, we studied the role of recombinant XIAP on EBI. Rats (n = 99) were allocated to the sham (n = 4), SAH+vehicle (n = 15) or SAH+r-XIAP (0.05 or 0.2 μg/rat, n = 40 in each group) groups. Body weight, neurological scores, BBB permeability, brain water content and expression of apoptosis-related proteins were detected 24 h after SAH (Fig. 1c).

Fourth, rats (n = 22) were allocated to sham (n = 6), SAH+ vehicle (n = 8) and SAH+r-XIAP (0.2 μg, n = 8) groups 1 h after SAH to study the role of XIAP on EBI post-treatment. Body weight, neurological scores, BBB permeability and brain water content were detected 24 h after SAH (Fig. 1d).

### ICV

The needle of a 10 μL Hamilton syringe (Hamilton Company, Reno, NV, USA) was inserted into the left lateral ventricle using the following coordinates relative to bregma^9^: 1.5 mm posterior, 1.0 mm lateral and 3.2 mm below the horizontal plane of bregma. Sterile PBS or human r-XIAP (0.05 or 0.2 μg in 1 μL of PBS) was infused at a rate of 0.5 μL/min (irrespective of the animal’s body weight) 1 h prior to surgery (as a pre-treatment) or 1 h after surgery (as a post-treatment). In the sham ICV rats, a burr hole was created on the skull at the same position, but no needle was inserted, and no drug infusion was given. The needle was removed 10 min after the infusion and the burr hole was quickly plugged with bone wax.

### Neurobehavioral Testing

Neurological deficits were blindly evaluated using a scoring system previously reported by Garcia *et al*., with slight modifications^10^. The minimum neurological score in this system is 3, and the maximum is 18. The scores were assessed by summing the scores of the 6 tests (spontaneous activity, spontaneous movement of 4 limbs, forepaw outstretching, climbing, body proprioception and response to whisker stimulation) according to previously described methods^11^. The beam balance test was used to investigate the animal’s ability to walk on a narrow wooden beam (2.25 cm in diameter and columnar) for 60 sec (4 points: walking ≥20 cm; 3 points: walking ≥10 cm but <20 cm; 2 points walking ≥10 cm but falling; 1 point: walking <10 cm; and 0 points: falling within walking <10 cm). We calculated the mean score of 3 consecutive trials in a 5-min interval. Two independent investigators who were blinded to the grouping recorded the measurements.

### Brain water content

The entire brain was removed and immediately weighed (wet weight) and then weighed again after drying in an oven at 105 °C for 24 h (dry weight). The percentage of brain water content was calculated using the following formula:


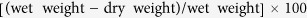


### BBB permeability

We assessed BBB permeability based on the protocol by Uyama *et al*.^12^ and the extraction method by Rossner and Temple^13^. Evan’s blue dye (2%; 5 mL/kg) was injected into the right femoral vein over 2 min and allowed to circulate for 60 min. The amount of extravasated Evan’s blue dye in the brain was determined using spectrofluorophotometry. Measurements were conducted at an excitation wavelength of 620 nm, an emission wavelength of 680 nm and a bandwidth of 10 nm.

### Quantitative real-time RT-PCR

Total RNA was extracted from brain samples using TRIzol reagent (Gibco BRL; Life Technologies, Rockville, MD, USA) according to the manufacturer’s instructions. The expression level of glyceraldehyde phosphate dehydrogenase (GAPDH) was evaluated as a specific internal control. To generate a standard curve, serially diluted standard plasmids were examined. The reaction mixture contained diluted cDNA, SYBR Green I Nucleic Acid Stain (Invitrogen Life Technologies, Carlsbad, CA, USA), 20 μM of each gene-specific primer and nuclease-free water to a final volume of 50 μl. The PCR reactions were cycled 40 times using a three-step cycle procedure (denaturation at 95 °C for 15 s, annealing at 60 °C for 1 min, and extension at 72 °C for 1 min) after two initial stages (45 °C for 2 min followed by 95 °C for 10 min). All samples were analyzed in triplicate.

### Western blotting analysis

Western blotting was performed as previously described. The samples (20 μg of protein) were separated using sodium dodecyl sulfate polyacrylamide gel electrophoresis with a 10% polyacrylamide gel. The following primary antibodies were used: goat monoclonal anti-caspase-3 (P17, 1:1000), caspase-8 (1:1500), caspase-9 (1:1000), AIF (1:1000), p53 (1:1500), Cyto-c (1:1000) and rabbit polyclonal anti-β-tubulin (1:3000) antibody (Santa Cruz Inc. USA). The nitrocellulose membranes were incubated with the primary antibodies and then washed with TBST buffer and incubated for 1 h at room temperature with the appropriate horseradish peroxidase-labeled secondary antibodies (1:1000, Santa Cruz Inc. USA) diluted in 1% nonfat milk in TBST. The membranes were rinsed twice and washed four times with PBS/Nonidet P-40 or TBST and then incubated with ECL (Amersham, Little Chalfont, UK) reagent for HRP (60 s) and exposed to autoradiography film to visualize the protein bands. The results were quantified using ImageJ software (NIH).

### TUNEL staining

Brain sections were stained using a TUNEL Staining Kit (Roche Inc., Basel, Switzerland) and the TUNEL-positive cells were expressed by fluorescein-dUTP with dNTP or POD (according to the manufacturer’s protocol for the *In Situ* Apoptosis Detection Kit [Roche Inc.]) based on previously described methods^14^. Sections were stained using a similar protocol, except the TUNEL reaction mixture was omitted as a negative control. Cells that showed nuclear condensation/fragmentation and apoptotic bodies in the absence of cytoplasmic TUNEL reactivity (green staining of nuclei) were considered apoptotic cells. Rabbit anibody to neuron (Santa Cruz, Shanghai China, 1:1500) with red fluorescence was used to lable neuronal cells. Apoptotic cells were confirmed with the help of a pathologist blinded to the grouping. The number of TUNEL-positive cells in each region was counted using a high-powered field (×400) by an investigator who was blinded to the grouping; the results were expressed as number/mm^2^.

### Cell death assay

A commercial enzyme immunoassay kit was used to quantify DNA fragmentation, which indicates apoptotic cell death, in brain tissue samples by determining cytoplasmic histone-associated DNA fragments (Roche Molecular Biochemicals, USA). The cytosolic protein fraction was extracted from fresh brain tissue according to previously described methods. A cytosolic volume containing 50 μg of protein was used for the enzyme linked immunosorbent assay according to the manufacturer’s instructions.

**Figure 2:**
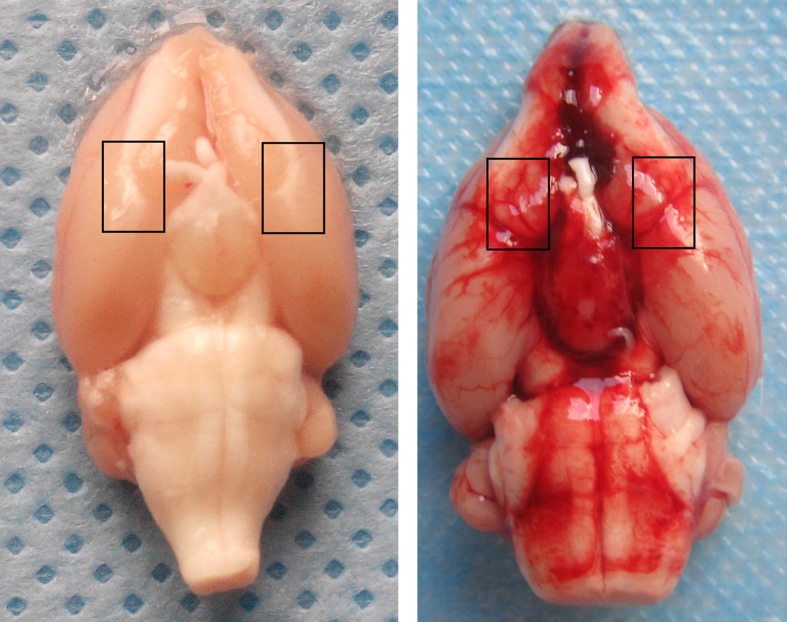
Brain cortex selected in the research. As shown in the picture, basal cortex adjacent to the blood were selected for Western blotting, TUNEL staining and real-time RT-PCR in the research.

The bilateral basal brain cortex adjacent to blood was selected for TUNEL staining, Western blot, PCR and cell death assay in the current research (Fig. 2).

### Statistical analysis

The data are expressed as the mean ± SD. Significant differences among the groups were compared using a one-way ANOVA, followed by Tukey-Kramer multiple comparison procedure if significant difference were observed in the ANOVA. The neurological and beam balance scores are expressed as median and the 25th–75th percentiles and were analyzed using the Mann-Whitney U test or Kruskal-Wallis test, followed by Steel-Dwass multiple comparisons. A probability value of *P < *0.05 was considered statistically significant.

## Results

### Physiological monitoring and the SAH model

Mean arterial blood pressure, blood gases and blood glucose were monitored before SAH induction. All animals were in the normal range prior to surgery (MABP, 80–110 mmHg; pCO_2_, 35–45 mmHg; blood glucose, 80–120 mg/dl). No animals died in the sham group, and 6 rats died within 6 h after blood injection.

**Figure 3:**
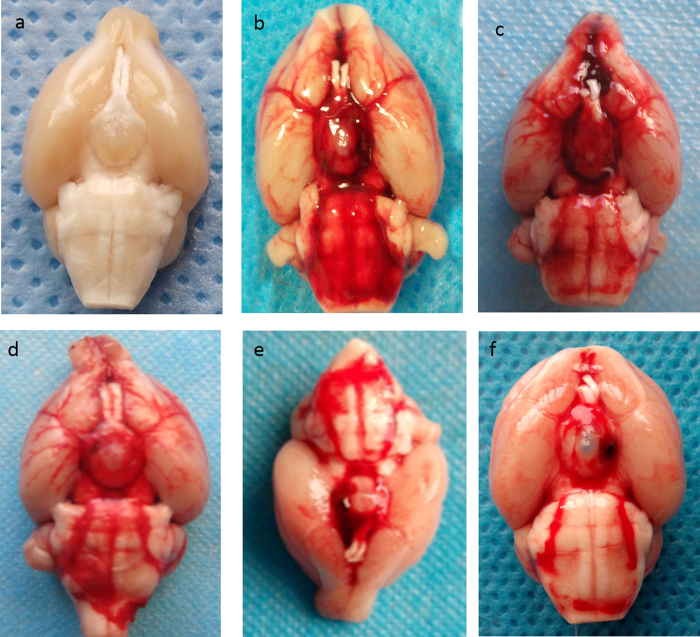
Represent picture of brain in each group. The rats were sacrificed and blood distribution was recorded according to the SAH grade method. (**a**) sham operation; (**b**) SAH control group; (**c**) SAH+scramble siRNA group; (**d**) SAH+XIAP siRNA group; (**e**) SAH+embelin group; (**f**) SAH+r-XIAP group.

The SAH grade was evaluated according to the score system reported previously^15^. The mean scores were 13.5 ± 2.3 in SAH control group, 13.8 ± 1.9 in SAH+scramble siRNA group, 12.9 ± 2.7 in SAH+XIAP siRNA group, 13.4 ± 2.1 in SAH + embelin group, 13.2 ± 1.8 in SAH + r-XIAP (0.05 μg) and 12.8 ± 2.1 in SAH + r-XIAP (0.2 μg) group. There was no obvious difference in SAH scores among groups.(*P* > 0.05). The represent picture of different group was shown in Fig. 3.

### XIAP expression after SAH

**Figure 4:**
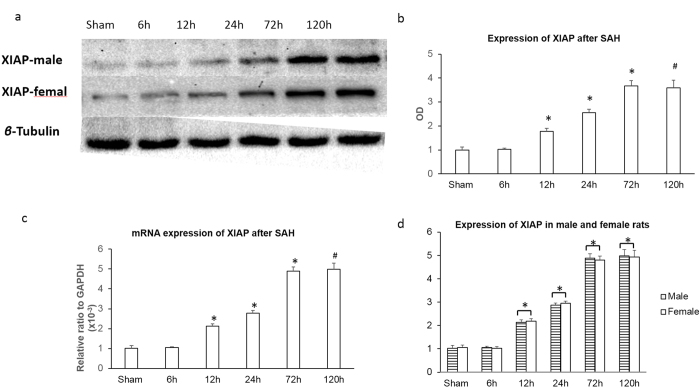
XIAP expression after SAH. XIAP mRNA and protein expression was assessed at 6, 12, 24, 72 and 120 h after SAH in both male and female rats. The basal level of XIAP expression was observed in the sham-operated control animals. XIAP was increased 12 h after SAH and reached its peak 72 h after SAH, with no further increase 120 h after SAH (*P < 0.05 compared with the sham and 6 h group and ^#^*P* > 0.05 compared with the 72 h group in (**a–c**). *P > 0.05 in d).

**Figure 5:**
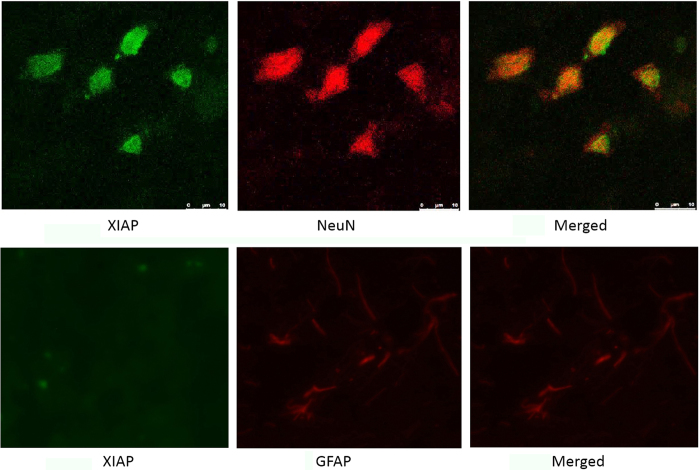
Distribution of XIAP after SAH. The distribution of XIAP in different cell types was examined using a confocal fluorescence microscope. The results showed that XIAP was mainly expressed in neuronal cells after SAH.

Real-time RT-PCR and western blotting were used to explore XIAP expression in two brain regions after SAH: the basal cortex adjacent to the injection site and the hippocampus. The bilateral basal frontal lobes were selected in this model [shown in Fig. 2]. XIAP expression was detected in normal control animals. In the cortex, XIAP was not significantly changed 6 h after SAH, was increased 12 h after SAH and peaked 72 h after SAH. A similar high level of XIAP expression was observed at 120 h compared to that at 72 h (Fig. 4a,b). The time course of XIAP expression in hippocampus was similar to the cortex. Real-time RT-PCR showed that XIAP mRNA expression was increased 6 h after SAH both in the cortex and the hippocampus, and reached its peak at 72 h after SAH, with no further increases at 120 h after SAH (Fig. 4c). We also tested the expression of XIAP in both male and female rats after SAH and found that there was no obvious difference in XIAP expression between male and female rats (Fig. 4a,d). Immunofluorescence was used to detect the distribution of XIAP expression. The results showed that XIAP was mainly expressed in neuronal cells in the basal cortex covered with thick SAH. There was no obvious expression of XIAP in the astrocytes (Fig. 5).

### Inhibition of endogenous XIAP on EBI after SAH

The siRNA efficiency was first examined *in vitro* using HEK293 cells. XIAP mRNA and protein expression were decreased by approximately 85% in cells transfected with siRNA-XIAP, whereas this reduction was only approximately 8.9% in cells transfected with scrambled siRNA (supplementary figure). The synthesized sequences of siRNA targeting XIAP were: 5′-UUUCUAUGUCAGUACAUGCUA-3′(sense); 5′-GCAUGUACUGACAUAGAAAAG-3′(sense). A scramble siRNA was used as negative control. The sequence of non-targeting control siRNA was 5′-AGUACUGCUUACGAUACGGdTdT-3′ (sense).

**Figure 6:**
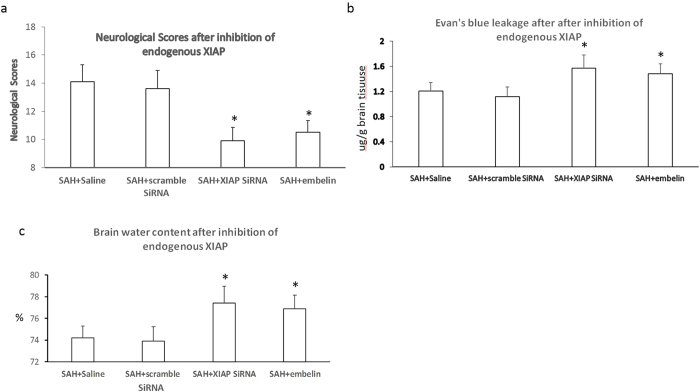
Effects of endogenous XIAP inhibition on EBI after SAH. The neurological scores, Evan’s blue dye leakage and brain water content were assessed after inhibition of endogenous XIAP using siRNA or embelin. The results showed that the neurological scores were further impaired and the Evan’s blue dye leakage and brain water content were increased (*P < 0.05 compared with the SAH and scrambled siRNA control group).

There were no obvious differences in body weight, blood gas or MABP among the groups. The neurological scores and BBB permeability was detected 72 h after SAH at the time point of peak XIAP expression. The neurological scores, brain edema and BBB permeability were similar in the animals treated with scrambled siRNA compared to the SAH group. The neurological scores were further impaired, and both the BBB permeability and brain water content were significantly increased in the animals treated with XIAP-siRNA and embelin compared with the animals treated with scrambled siRNA and the saline control animals (Fig. 6).

### Exogenous XIAP treatment

There were no differences in the physiological parameters among the SAH groups. The neurological scores and BBB permeability were detected 24 h after SAH at a time point prior to the severe impairment of BBB permeability and when the expression of XIAP was not at its highest level to exclude the role of endogenous XIAP.

**Figure 7:**
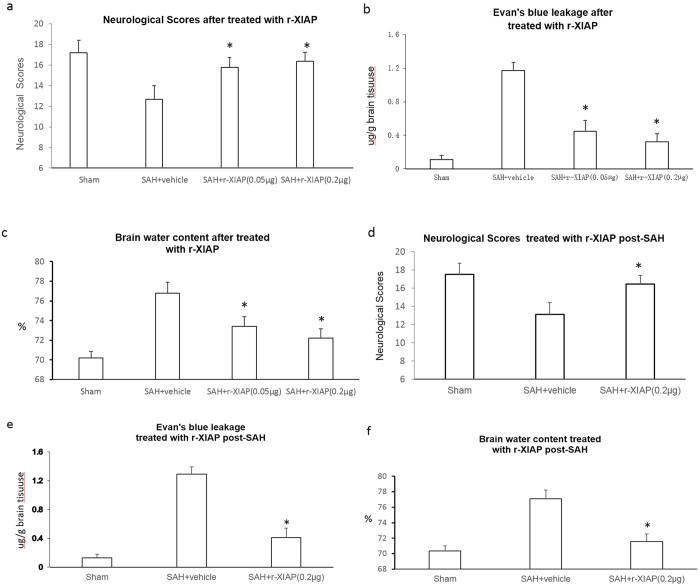
Role of r-XIAP on EBI after SAH. The neurological scores, Evan’s blue dye leakage and brain water content were assessed after r-XIAP treatment. The results showed that r-XIAP improved the neurological scores and decreased the brain water content and leakage of Evan’s blue dye (**a–c**) *P < 0.05 compared with the SAH control group). Treatment with r-XIAP 1 h post-SAH showed the same effects of ameliorating EBI (**d–f**) *P < 0.05 compared with the SAH control group)

The neurological scores were increased (15.78 ± 1.00 in the 0.05 μg group and 16.36 ± 0.98 in the 0.2 μg group vs 12.67 ± 1.34 in SAH+ vehicle group), the leakage of Evan’s blue was decreased (0.45 ± 0.13 μg/g brain tissue in the 0.05 μg group and 0.32 ± 0.10 μg/g brain tissue in the 0.2 μg group vs 1.17 ± 0.18 μg/g brain tissue in the vehicle group) and brain water content was decreased (73.4 ± 1.01% in the 0.05 μg group and 72.2 ± 0.98% in the 0.2 μg group vs 76.8 ± 1.10% in the vehicle group) after treatment with r-XIAP (Fig. 7a–c).

We also examined the effects of r-XIAP treatment on EBI when it was administered 1 h after SAH induction. The results showed that EBI was ameliorated in the r-XIAP-treated rats post-SAH compared with the vehicle-treated group (Fig. 7d–f).

### TUNEL staining and cell death assay

**Figure 8:**
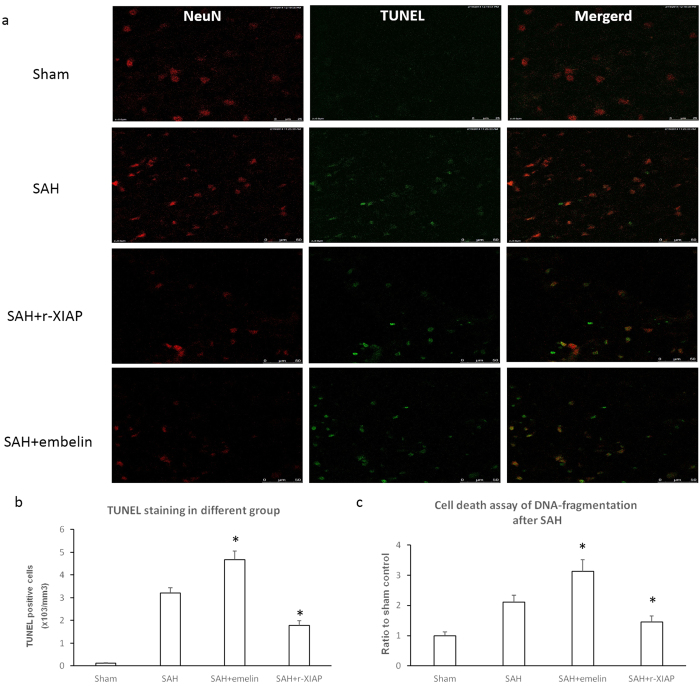
TUNEL staining and cell death assay. TUNEL staining and cell death assay were used to detected apoptosis in the different groups after SAH. The results showed that embelin increased apoptosis and DNA fragmentation, whereas r-XIAP decreased apoptosis and DNA fragmentation. Representative TUNEL staining images of different groups is shown in Fig. 8a (*P < 0.05 compared with the SAH control group).

The results showed that r-XIAP decreased apoptosis, whereas embelin increased apoptosis after SAH in neuronal cells (Fig. 8a,b). DNA fragmentation was decreased in the r-XIAP-treated rats and increased in the embelin-treated rats (Fig. 8c).

### Expression of apoptosis-related proteins

**Figure 9:**
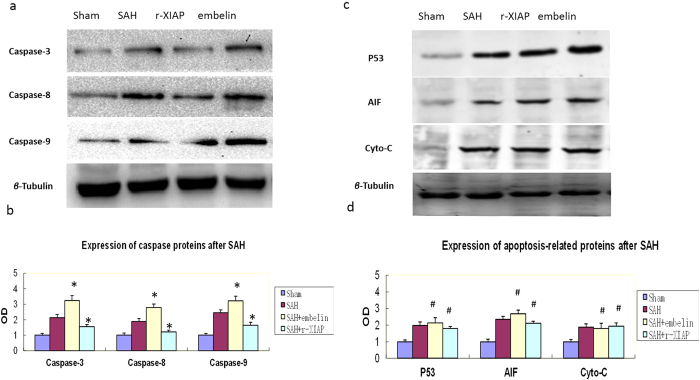
Expression of apoptosis-related proteins after SAH. The expression of caspase-3, -8 and -9 was detected in the different groups. The results showed that r-XIAP inhibited the expression of caspase-3, -8 and -9 and had no obvious effects on p53, AIF and cytochrome c. Embelin further increased the expression of caspase-3, -8 and -9 and had no obvious effects on p53, AIF and cytochrome c (*P < 0.05 compared with the SAH control group; ^#^*P* > 0.05 compared with the SAH control group).

The results showed that caspase-3, caspase-8 and caspase-9 were up-regulated after SAH. The expression levels of caspase family proteins were further increased in the embelin-treated rats and decreased in the rats treated with r-XIAP (Fig. 9a,b). The levels of AIF, p53 and cytochrome c were increased after SAH; however, no changes in these proteins were observed following embelin or r-XIAP treatment (Fig. 9c,d).

## Discussion

In this study, we observed that XIAP expression was increased after SAH, mainly in neuronal cells. The inhibition of XIAP using either siRNA transfection or embelin increased apoptosis and aggravated EBI. Recombinant XIAP was administered both pre- and post-SAH to explore the effects of XIAP on EBI. The results showed that r-XIAP ameliorated EBI and preserved the BBB. At the same time, apoptosis-related proteins were detected. These results suggest that XIAP may ameliorate EBI by acting on the caspase-dependent apoptosis pathway.

Early brain injury (within 72 h after SAH) is one of the most important factors that determines the prognosis of patients following intracranial aneurysm rupture^3^,^4^,^16^. Apoptosis has been found to play a key role in EBI and inhibiting apoptosis resulted in neuroprotective effects in an experimental model of SAH^14^,^17^,^18^. Inhibitors of apoptosis (IAPs), especially XIAP, are the most potent endogenous inhibitors of apoptosis and have a high efficiency and affinity with executor and/or trigger caspases^19^. Six members of the IAP family of proteins have been found to date, including XIAP, Bruce, NIAP and Survivin^6^,^19^. The IAPs have a caspase-recruitment domain and an N-terminal baculoviral inhibitor-of-apoptosis repeat motif, which is necessary for biological activity. IAPs contain a C-terminal RING zinc finger domain that is involved in protein-protein and protein-nucleic acid interactions^20^. Through direct inhibition of caspase-3 and caspase-9, XIAP modulates the Bax/cytochrome c pathway by inhibiting caspase-9^21^.

The role of XIAP in neuronal survival or death after SAH has not been previously examined. In the present study, XIAP was increased after blood injection into the subarachnoid space. The inhibition of endogenous XIAP further aggravated EBI after SAH, and r-XIAP treatment had neuroprotective effects. These results indicated that the overexpression of XIAP may have anti-apoptosis effects. The present results demonstrate that the up-regulation of XIAP may be a self-protective mechanism that prevents early brain injury after SAH. Furthermore, XIAP did not affect the normal apoptotic procedure; therefore, it may be a safer method of preventing apoptosis compared with selective apoptosis inhibitors. Based on the present results, XIAP may participate in the regulation of apoptosis after SAH and targeting XIAP may be a potential method to prevent EBI after SAH.

It was reported that XIAP mainly interacted with caspases proteins during apoptosis^22^. In the current researh, expression of caspase-3,caspase-8 and caspase-9 was inhibited by r-XIAP after SAH. We also detected expression of cytochrome C, P53, and AIF after SAH. It was found that all these apoptosis-related proteins were up-regulated after SAH. This was consistent with previous reports^3^,^23^ and our previous published results^14^. No obvious changes were observed of these proteins after treated with r-XIAP and embelin, the reason may be due to XIAP has no combined domain with these proteins. This might be the superiority of XIAP in anti-apoptosis due to its effects on caspases-dependent pathway and with no effects on other apoptosis-related prteins.

It has been reported that XIAP plays a different role in male and female rats following brain ischemia and that XIAP ameliorates brain injury after MCAO in female rats^24^. In a traumatic brain injury model, XIAP expression was different between male and female rats^25^. In the current study, XIAP expression was detected in both male and female rats; however, no significant difference in XIAP expression was found between male and female rats after SAH. One reason for the differential results may be attributed to the SAH model we used. Autologous blood injection induced apoptosis in neuronal cells and stimulated XIAP expression in both male and female rats.

Role of microglia in EBI following SAH has been reported previously^26^,^27^,^28^,^29^.Inhibition of activation of microglia via mTOR pathway has the effects of decreasing EBI after SAH^26^. It was reported that Ethyl pyruvate alleviating early brain injury following SAH through inhibiting of microglia activation^27^. At the same time, microglia has the role of regulating blood and heme clearance, which may be pivotal in neuronal injury and cognitive dysfunction after SAH^28^. Except for early brain injury, it has been found that microglia also inflicts delayed brain injury after SAH, which may be important factors in determining the long-term prognosis of SAH patients^29^.

It has been reported that XIAP was expressed in inflammatory microglia under stimulation with erythropoietin (EPO)^30^. Whether XIAP expression was changed after SAH has not been studied previously. In our current study, we mainly researched the expression of XIAP in neuron. It deserves investigation in the future work that if microglia express XIAP after SAH.

The endothelium and tight junction proteins are the main structural components of BBB. Apoptosis of endothelial cells occurred after experimental SAH in rats, and inhibition of apoptosis of endothelial cells has the role of protection of BBB^4^,^31^,^32^. In the present research, protection of BBB treated with r-XIAP was observed, as it was shown in decrease of brain water content and Evan’s blue leakage. Whether protection of BBB was through inhibition of apoptosis of endothelial cells was not detected based on our results. The neurological improvement of the SAH rats treated with r-XIAP was associated with inhibition of neuronal apoptosis and preservation of BBB, and the mechanisms related to BBB protection of XIAP were not further investigated in the present research. We mainly detected the apoptosis of neuronal cells in the current study. It deserves investigation in the future whether XIAP has the effects of inhibition apoptosis of endothelial cells after SAH. Several SAH models have been used to detect pathophysiological changes after SAH, including the perforating middle cerebral artery model, autologous blood injection into the cisterna magna model and autologous blood injection into prechiasmatic cistern model^33^,^34^. The advantages and disadvantages of each model have been discussed in previous reports^35^. In the current study, autologous blood was injected into the prechiasmatic cistern. This model has the advantage of low mortality and the severity of hemorrhage was similar among the rats. Furthermore, previous studies have shown that obvious disruption of the BBB and apoptosis occur in this model^36^. A hemorrhage scoring system was used to evaluate the severity of hemorrhage on necropsy according to previously reported methods^15^. The prechiasmatic cistern blood injection SAH model is one of the most used models in studying early brain injury following SAH^36^,^37^,^38^. It has the advantage in reproducibility and low morbility. The early brain injury, as indicated by increasing of brain water content, disruption of BBB, decreasing of neurological scores and apoptosis of neuronal cells was observed in our experiment and other reports^36^,^37^,^38^. The results showed that there was no significant difference in the mean hemorrhage scores among the groups. This indicated that the differences in brain edema, BBB permeability and neurological scores was due to XIAP inhibition or r-XIAP treatment.

## Conclusions

The present results show that XIAP was increased and played a protective role against EBI after SAH. The effects of XIAP were associated with inhibition of the caspase-dependent apoptosis pathway. Targeting XIAP may improve the prognosis of SAH patients.

## Additional Information

**How to cite this article**: Cheng, G. *et al*. X-linked inhibitor of apoptosis inhibits apoptosis and preserves the blood-brain barrier after experimental subarachnoid hemorrhage. *Sci. Rep.* **7**, 44918; doi: 10.1038/srep44918 (2017).

**Publisher's note:** Springer Nature remains neutral with regard to jurisdictional claims in published maps and institutional affiliations.

## Supplementary Material

Supplementary Information
