# Supplementary material for: X-linked inhibitor of apoptosis inhibits apoptosis and preserves the blood-brain barrier after experimental subarachnoid hemorrhage
Source: Sci Rep. 2017 Mar 22;7:44918. doi: 10.1038/srep44918 (PMC5361183; doi:10.1038/srep44918)
Supplement: Supplementary Information [file srep44918-s1.pdf]

Supplementary Figure

X-linked inhibitor of apoptosis inhibits apoptosis and preserves the blood-brain barrier after experimental subarachnoid hemorrhage

[1*]Gao Cheng, [1#]Yu Hongwei, [1]Yan Cong, [1]Zhao Wenyang, [1]Liu Yao, [1]Zhang Dongdong, [1]Li Jingwei, [1]Liu Nan

1,Department of Neurosurgery, The First Affiliated Hospital of Harbin Medical University, Harbin, Heilongjiang, China

*Correspondence to gaocheng7730@163.com

[#]Yu contributed equally to this paper.

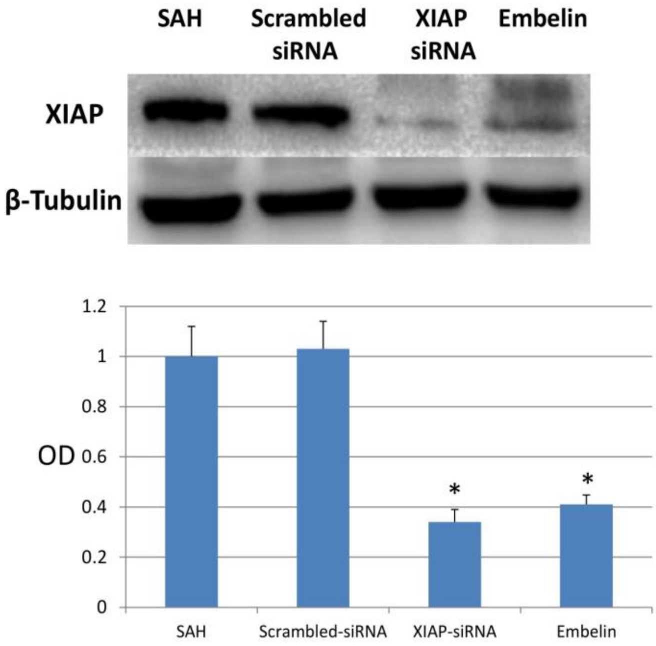

Supplemental Figure. Inhibition of XIAP expression by XIAP-siRNA and embelin. XIAP expression was detected at 72 h after SAH, the results showed that XIAP was inhibited by XIAP and embelin obviously. *,P<0.05 versus SAH and scrambled group.
